# DC Microplasma Jet for Local a:C-H Deposition Operated in SEM Chamber

**DOI:** 10.3390/mi8070211

**Published:** 2017-07-03

**Authors:** Khanit Matra, Hiroshi Furuta, Akimitsu Hatta

**Affiliations:** 1 Department of Electrical Engineering, Engineering Faculty, Srinakharinwirot University, Bangkok 10110, Thailand; khanit@g.swu.ac.th; 2 Department of Electronic and Photonic Systems Engineering, Kochi University of Technology, Tosayamada-cho, Kami, Kochi 782-8502, Japan; furuta.hiroshi@kochi-tech.ac.jp; 3 Center for Nanotechnology, Research Institute of Kochi University of Technology, Tosayamada-cho, Kami, Kochi 782-8502, Japan

**Keywords:** micro plasma jet, C_2_H_2_ plasma, thin film deposition, SEM

## Abstract

A DC micro plasma jet for local micro deposition of a:C-H film in the ambient vacuum of scanning electron microscope (SEM) chamber is proposed. Acetylene (C_2_H_2_) gas was locally fed into the chamber through an orifice shaped gas nozzle (OGN) at 6.6 sccm in flow rate by applying 80 kPa-inlet pressure with an additional direct pumping system equipped on the SEM chamber. As a cathode, a cut of n-type silicon (Si) wafer was placed right in front of the OGN at 200 μm gap distance. By applying a positive DC voltage to the OGN, C_2_H_2_ plasma was generated locally between the electrodes. During discharge, the voltage increased and the current decreased due to deposition of insulating film on the Si wafer with resulting in automatic termination of discharge at the constant source voltage. A symmetric mountain-shaped a:C-H film of 5 μm height was deposited at the center by operation for 15 s. Films were deposited with variation of gas flow rate, gap distance, voltage and current, and deposition time. The films were directly observed by SEM and analyzed by surface profiler and by Raman spectroscopy.

## 1. Introduction

Thin film deposition has played an important role in many industrial applications such as semiconductor devices, electronic components, and tribological coating [1,2,3]. Among many methods for thin film deposition—for example: chemical vapor deposition (CVD) [4], spin coating [5], and thermal evaporation [6]—micro plasma jet has been promoted as a special technique for micro deposition owing to its many advantages such as high plasma density, fast convection flow, miniature geometry, and flexible operation [7]. Micro plasma jet is hence one interesting tool for chemical deposition, recently. Moreover, micro plasma jet could be also applied in surface modifications, analytical chemistry, nanostructure growth, micro chemical reactions, and biomedical treatment among others [1,2,3,7,8]. 

In the past decade, we have been developing micro plasma jet operated under the vacuum environment in the scanning electron microscope (SEM) chamber. Our target of micro plasma processing in SEM is to perform local restoration of microelectronic devices such as large-scaled integrated circuits (LSIs), fabrication of micro-electromechanical systems (MEMS) devices, and growth of nanostructured material with in situ SEM observation. The advantages of micro plasma processing in SEM are easy ignition of discharge by electron beam and in situ observation of material surface. 

For operation of gas discharge in SEM chamber, however, it is necessary to supply sufficient pressure of discharge gas while the ambience should be kept at high vacuum for SEM operation. The solution is a micro sized high pressure gas jet with a small gas flow rate. Due to the small gas flow rate and an additional vacuum pumping system, the ambient vacuum pressure can be kept low enough for normal SEM operation. If a capillary shaped gas nozzle is used, the pressure at the exit of the gas nozzle will be fairly decreased due to a small conductance of the capillary. Instead, by using an orifice shaped gas nozzle (OGN), as a small hole on a thin wall, a higher pressure gas jet will appear locally at the exit of the orifice. By applying a high pressure to the micro-sized orifice, a high dense gas jet of a micro diameter can be emitted into the vacuum environment [9]. 

In this paper, how the micro plasma jet CVD works as a local thin film deposition technique in SEM is demonstrated. The characteristics of electrical discharge in micro plasma jet and the properties of deposited films were studied with variation of electrode gap distance, discharge current, discharge voltage, and deposition time. Deposited thin-films were examined using in situ SEM observation, surface profiler, and Raman spectroscopy. 

## 2. Experimental Apparatus and Procedure

**Figure 1:**
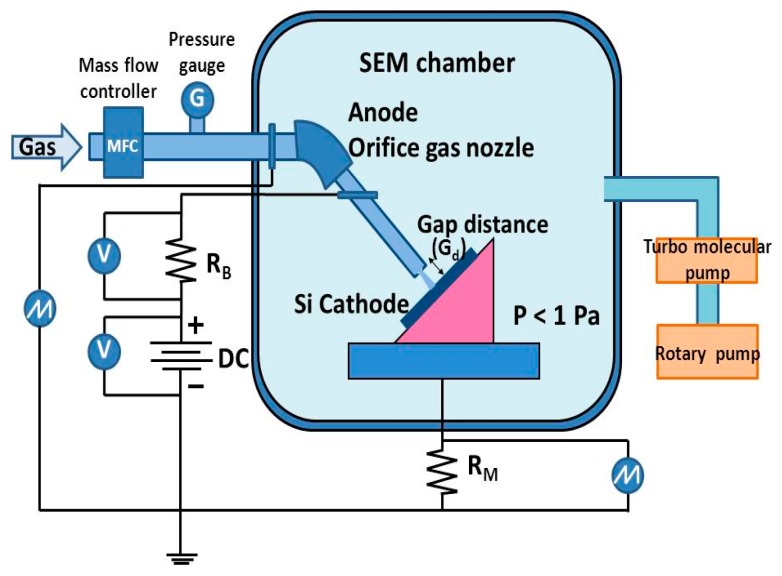
A schematic diagram of micro plasma jet set up in SEM.

The experiments in this research were performed inside SEM (S-3000N, Hitachi High-Technologies Corporation, Tokyo, Japan) chamber as shown in Figure 1. The OGN as the anode and a cut of silicon (Si) wafer as cathode were supported on each 3-D micro-manipulator for precise positioning to each other and to the electron beam in SEM. The OGN and the Si wafer were vertically aligned with a gap distance (*G_d_*) ranging from 100 to 200 μm. The arrangement of electrodes was confirmed by SEM observation. 

**Figure 2:**
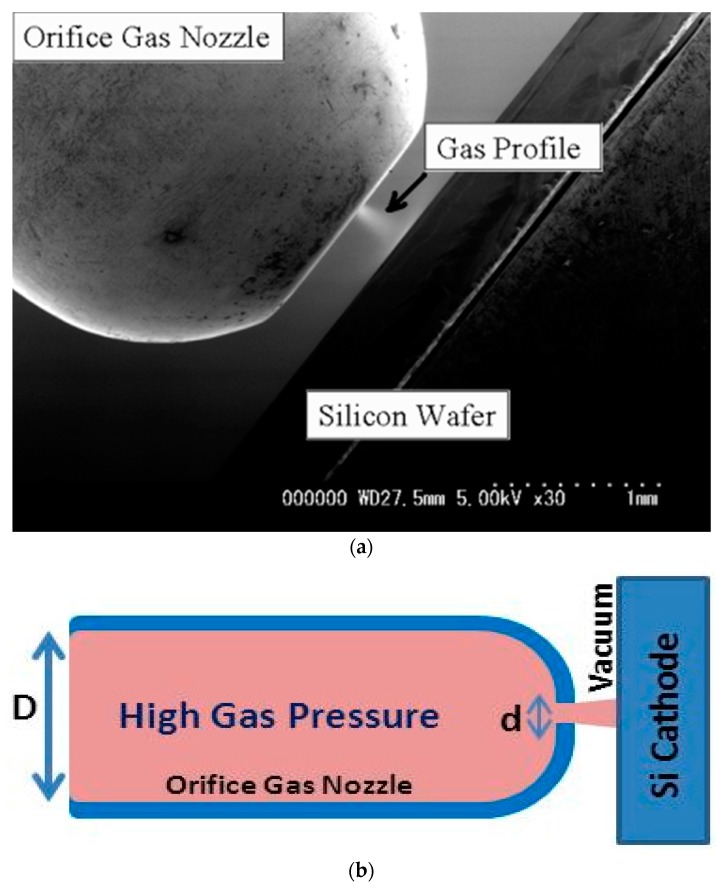
(**a**) An SEM image for the OGN and Si wafer with an image of C_2_H_2_ micro gas jet and (**b**) a schematic drawing for OGN.

An SEM image for OGN, Si wafer, and injected gas jet is shown in Figure 2a. The injected gas profile can be seen in the SEM image when the gas density as the local pressure is high enough, for instance, nearly atmospheric pressure. Because the gas molecules can be ionized by probe electron beam with resulting in electron emission from the molecules, the image of gas profile appears in the same way as the conventional secondary electron emission (SE) image from solid surface. The brightness of the gas image shows the gas density, i.e., local pressure in the gas jet. 

For gas discharge and CVD, acetylene (C_2_H_2_) gas was supplied into the gap through the OGN as shown in Figure 2b. The OGN was manufactured by Lenox Laser, Inc. A standard 1/8′′ SS-316 tubing was closed at one end and a laser-drilled hole of 30 μm diameter (*d* = 30 μm) was opened on the thin wall of 250 μm in thickness (SS-1/8-TUBE-30). By using Orifice Calculator^©^ from the manufacturer, the expected gas flow rate of the 30 μm orifice for C_2_H_2_ at 300 K is 8 sccm when the inlet pressure is 100 kPa and the outlet pressure is 0 (vacuum). Because the inner diameter of tube (*D* = 3.05 mm) is sufficiently larger than the orifice, the pressure in the tube can be kept almost constant until the exit of orifice and the high pressure gas jet can be emitted at the exit of orifice. The C_2_H_2_ gas was supplied through a mass flow controller (STEC INC, SEC-E40) at 6.6 sccm flow rate and the inlet pressure monitored by a pressure gauge (TP-618B, Tokyo Aircraft instrument Co. Ltd., Tokyo, Japan) was about 80 kPa. To maintain the vacuum level in the SEM chamber below 1 Pa which is the system requirement for normal operation, a turbo molecular pump (PFEIFFER VACUUM, TMU071YP) was additionally equipped directly on the SEM chamber. 

The pressure in the SEM chamber can be estimated from the balance between supplied gas flow rate and the pumping speed of TMP while the out gas from the chamber has been evacuated through the conventional vacuum system of SEM. The TMP was installed on the SEM chamber through a port 40 mm in diameter. The effective pumping speed of TMP is 33 L/s (N_2_) as equivalent to the spec of the product with an option of DN40 connection port. The increase of pressure in the chamber (on the chamber wall and in front of the TMP connection port) for 1 sccm gas flow is found as 0.05 Pa dividing by the pumping speed of 33 L/s where 1 sccm = 100 kPa∙1 mL/60 s = 1.67 Pa∙L/s. Supposing the same pumping rate for C_2_H_2_ gas, the chamber pressure was increased to 0.33 Pa at the experimental condition of a 6.6 sccm flow rate. It requires numerical simulation for understanding the detail gas profile discharge in the vicinity of OGN. As indicated later, however, a high density gas jet was observed as an SEM image only in the vicinity of OGN.

The micro plasma discharge was excited using a DC high voltage source of 1 kV maximum. A positive high voltage was supplied to the OGN as an anode through a large ballast resistor (*R_B_* = 10 MΩ) to limit the current below 100 μA. For DC characteristics, the voltages of power source and ballast resistor were measured using digital multi meters. The DC discharge current (*I_d_*) was obtained from the voltage drop across *R_B_* and the DC discharge voltage (*V_d_*) was obtained from the difference between the source voltage and the voltage drop across *R_B_*. The discharge was not always stable but sometimes pulsed even if the DC voltage was supplied. For investigation of the self-pulsing discharge, the temporal change of current and voltage were monitored using a digital oscilloscope (LT364, Teledyne LeCroy Japan, Tokyo, Japan). The current waveform *i*_d_(*t*) was detected by a voltage drop across a resistance (*R_M_* = 100 kΩ) inserted between the cathode and ground, and the voltage waveform *v*_d_(*t*) was directly measured using a high voltage probe. For self-pulsing discharge, besides the temporal waveforms of current and voltage, DC current and voltage were measured as the time averaged. 

Because the current and voltage characteristics gradually drifted with discharge duration in a time scale of a few seconds due to film deposition on the Si wafer as cathode, a short duration of discharge was operated with monitoring the current and voltage waveforms. For the short discharge, an intentionally pulse modulated voltage—0.2 Hz frequency, 0.1% duty cycle, and 5 ms duration—was applied. 

## 3. Experimental Results and Discussion

The experiments in this research were performed inside SEM (Hitachi S-3000N) chamber as shown in Figure 1. The OGN as the anode and a cut of silicon (Si) wafer as cathode were supported on each 3-D micro-manipulator for precise positioning to each other and to the electron beam in SEM. The OGN and the Si wafer were vertically aligned with the gap distance (*G_d_*) ranging from 100 to 200 μm. The arrangement of electrodes was confirmed by SEM observation. 

### 3.1. Voltage and Current Characteristics of C_2_H_2_ Plasma Jet in Vacuum

**Figure 3:**
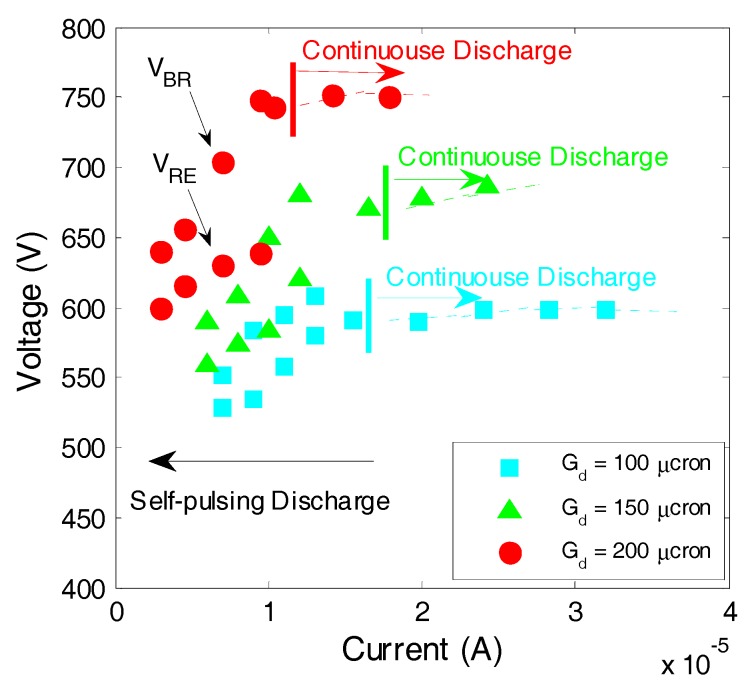
Current and voltage characteristics for micro plasma jet with variation of gap distance. For self-pulsing discharge, both the breakdown voltage (*V*_BR_) and recovery (sustain) voltage (*V*_RE_) are plotted.

**Figure 4:**
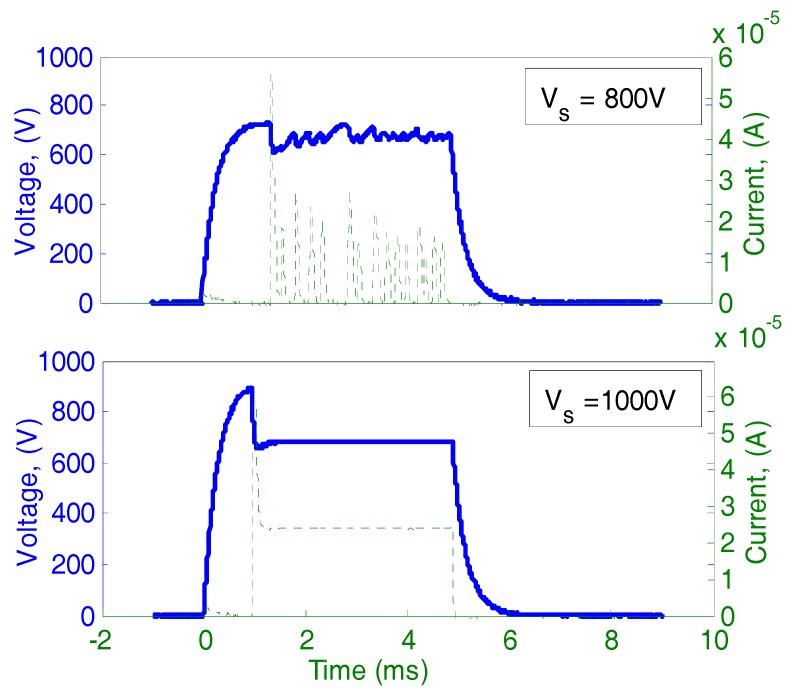
Current and voltage waveforms for intentionally shortened (5 ms) discharge duration with fresh Si wafer at 150 μm gap distance and 6.6 sccm gas flow rate, as self-pulsing at 800 V and stable DC at 1000 V.

Figure 3 shows the current and voltage plots and Figure 4 shows typical waveforms of voltage and current during an intentionally modulated pulse voltage at 6.6 sccm C_2_H_2_ flow rate (about 80 kPa inlet pressure). As shown in Figure 4, the discharge was automatically pulsing even if the source voltage was continuously applied at 800 V while it was quite stable at 1000 V. The two different types of discharge modes—self-pulsing mode and continuous mode—are similar to those in previous reports [10,11,12]. In our previous work, it had been confirmed that the continuous discharge mode was suitable for plasma processing such as micro sputter etching due to their stability [13]. 

Figure 3 shows current and voltage characteristics with a variation of gap distance from 100 to 200 μm. Two discharge modes are separated by each threshold of minimum discharge current for sustaining continuous discharge (*I_min_*), which is depicted by short thin vertical lines. The threshold current depends on the gas type, pressure and gas flow rate, electrode gap distance, electrode material, and other conditions. In the experimental results, the thresholds were 15.5, 16, and 11 μA for the gap distance of 100, 150, and 200 μm, respectively. At a current above the threshold, the discharge voltage was almost constant as expected for normal glow discharge scheme. By increasing the source voltage while the discharge voltage was almost constant, the discharge current increased as the voltage drop of the ballast resistor increased. In Figure 4, typical waveforms of current and voltage at 150 μm gap distance are shown. The required threshold current for continuous discharge was not supplied through the ballast resistor when the source voltage was 800 V while a sufficient current was supplied when the source voltage was increased to 1000 V. 

The self-pulsing discharge is characterized by breakdown voltage (*V_BR_*) and recovery voltage (*V_RE_*). The recovery voltage is the minimum and the breakdown voltage is the maximum in the voltage waveform during self-pulsing discharge which are required for sustaining and igniting discharge, respectively [13]. The voltage waveform oscillates between these two voltages. 

The self-pulsing of discharge is induced in an RC circuit consisting of the ballast resistor and a stray capacitor of anode wiring. The detailed explanation of self-pulsing discharge was explained in literature [12]. The current from the voltage source is limited by the large ballast resistor. The current limit depends on the resistance value and on the voltage drop as the difference between voltage source and required discharge voltage. When a discharge started at the breakdown voltage with insufficient current supply through the ballast resistor, only for a short duration, the gas discharge can be continued by the current from the stray capacitor. By discharging the stray capacitor, however, the anode voltage quickly dropped, resulting in automatic termination of gas discharge. In turn, after termination, the stray capacitor is charged again through the ballast resistor until the voltage reaches the breakdown voltage. 

**Figure 5:**
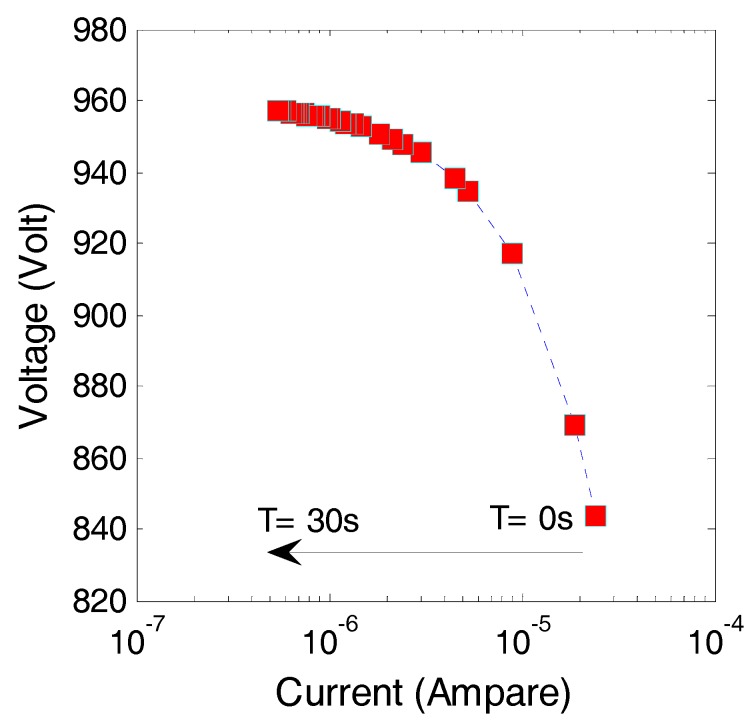
DC (time averaged) current and voltage plots by sampling every 0.5 s for 30 s in total at the electrode gap distance of 150 μm and at a continuous source voltage of 1000 V.

Due to deposition of film from C_2_H_2_ source gas on the Si wafer as cathode, the voltage and current characteristic drifted with discharge and deposition duration. Figure 5 shows the DC (time averaged) current and voltage plots by sampling every 0.5 s for 30 s in total at the electrode gap distance of 150 μm and at a continuous source voltage of 1000 V. The C_2_H_2_ discharge was automatically terminated after a few tens of seconds from the ignition of discharge with a virgin Si wafer. At this experimental condition, C_2_H_2_ plasma started at 845 V with a current of 24 μA. The continuous discharge mode was sustained only for a few milliseconds after ignition and quickly turned to the self-pulsing discharge mode. With the discharge and deposition, the voltage increased and the current decreased. After 15 s of discharge, the voltage increased to 955 V while the current decreased to 1.5 μA. 

**Figure 6:**
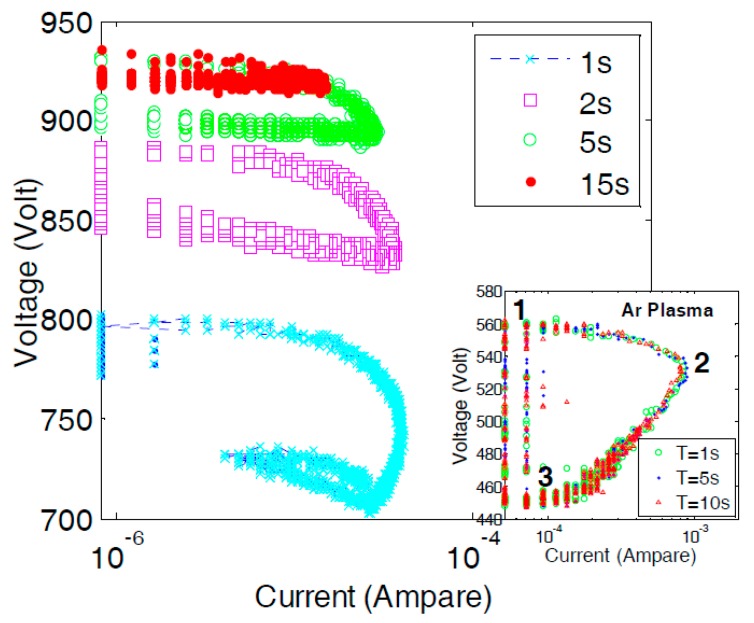
Plots of instantaneous current and voltage during self-pulsing discharge at different elapsed time, 1, 2, 5, and 15 s from ignition of C_2_H_2_ plasma at the electrode gap distance of 150 μm and the continuous source voltage of 1000 V. The inset is for Ar plasma jet at elapsed time 1, 2, and 5 s.

Figure 6 shows the plots of instantaneous current and voltage during self-pulsing discharge at different elapsed time, 1, 2, 5, and 15 s from ignition of C_2_H_2_ plasma at the electrode gap distance of 150 μm and the continuous source voltage of 1000 V. 

The inset in Figure 6 shows the plots of instantaneous current and voltage during self-pulsing discharge with Ar plasma jet at elapsed time 1, 2, and 5 s from ignition. The self-pulsing mode of Ar plasma jet could be separated into three phases per cycle [9,14]. During the self-pulsing, each pulse discharge started at the breakdown voltage and almost negligible current (mostly due to electric noise), as indicated “1” in the inset figure, the current increased to the peak, as indicated point “2”. During the discharge from point “1” to point “3”, the anode voltage dropped by discharging the stray capacitor to continue the instantaneous discharge. The discharge automatically terminates at the minimum sustain voltage (recovery voltage), as indicated point “3”, and the stray capacitor is charged until the next breakdown at point “1”. In the case of Ar discharge, the cycles of self-pulsing discharge repeated in the same way for a long duration. In the case of C_2_H_2_ plasma jet, the plots of self-pulsing cycle gradually shifted to the higher voltage side due to an increase in both breakdown voltage and sustain voltage. 

It was confirmed that the deposited film was highly insulated due to charge up of the surface during SEM observation. The effect of the insulating cathode surface on the discharge property is a drawback of DC gas discharge [15]. Moreover, local heating and arcing will result in degrading film properties and formation of micro particle on the surface [16]. Insulating film deposition on the electrode strongly affected the gas discharge properties not only by blocking the current flow but also by substitution of surface properties [17]. 

When the film thickness is still thin enough to permit a small amount of current flow in the film bulk, the deposited film simply performs as a series resistance. Due to the voltage drop that appeared in the film bulk, the apparent working voltage will increase. By increasing the operation duration, the film thickness will increase and the voltage drop will also increase. Due to increase of the voltage drop in the film as the series resistance, the voltage for the ballast resistor decreased instead at the constant source voltage and even at the constant gas discharge voltage. In the experiments, after a few milliseconds of operation, the discharge mode turned to the self-pulsing because the voltage for the ballast resistor decreased, resulting in a severe current limit. 

In the case of this micro plasma jet, it is also important to consider the local profile of film deposition. Due to the extreme gas profile, as shown in Figure 2a, the gas discharge current pass and the excited plasma profile will be localized only in the high density gas jet at first. As mentioned in the next section, the film deposition is also limited in the local area irradiated to the high pressure gas spraying. When the high pressure gas spraying area is fully covered with insulating film, it cannot work as a cathode and gas discharge should be sustained in the surrounding area where the gas density dramatically decrease by rapid diffusion into the vacuum environment. Moreover, the pass length from the anode to the working cathode area becomes much longer than the configured gap length. Due to an increase of the actual discharge pass length and the dramatic decrease of gas pressure on the working cathode area, the breakdown voltage and sustain voltage increase substantially. A possible solution for sustaining discharge through the insulating film is using AC, RF, and pulse DC power [7,16,17,18].

### 3.2. Properties of Deposited Thin Films 

On the Si wafer as cathode, an insulating film was deposited during operation of the C_2_H_2_ plasma jet. The reactions at the growing film surface occurred as a result of interactions between the silicon substrate and the ions, radicals, atoms, and gas molecules in the plasma [19,20,21]. For analysis on film properties, films were deposited at the gap distance of 100 and 200 μm, C_2_H_2_ flow rate of 6.6 sccm, inlet gas pressure of 80 kPa, and source voltage of 1000 V. 

**Figure 7:**
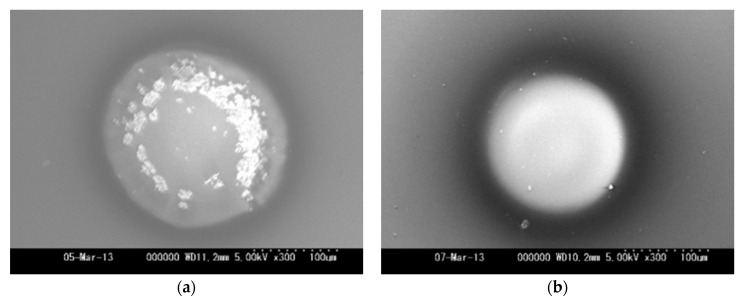
SEM images of cathode (Si) surfaces after 5 s operation at 1000 V source voltage, 6.6 sccm C_2_H_2_ flow rate, 80 kPa inlet pressure, and the electrode gap distance of (**a**) 100 and (**b**) 200 μm.

Figure 7a,b show the SEM images of Si wafers as cathode after 5 s operation at 100 and 200 μm in gap length with 38 and 19 μA of initial current, respectively. As shown in Figure 7a, there were many clusters or flakes on the film in the case of the 100 μm gap length. In the case of the 200 μm gap length, the surface looked almost smooth. Though the detail structure of the flakes has not clarified yet, the local high electric field would induce focusing of current with resulting in breaking films and formation of micro particles [16]. 

**Figure 8:**
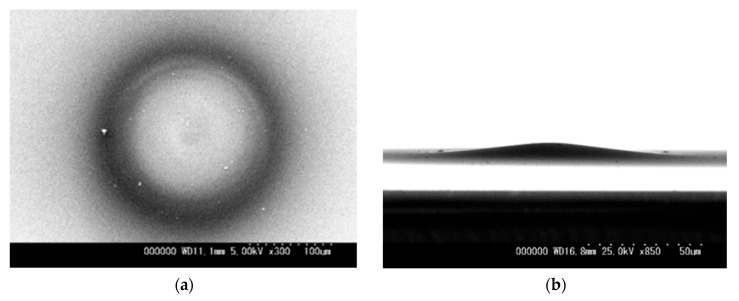
SEM image from (**a**) top view and (**b**) side view of deposited film after 15 s operation at 200 μm gap length, 6.6 sccm C_2_H_2_ flow rate with 80 kPa inlet pressure, and 1000 V source voltage.

**Figure 9:**
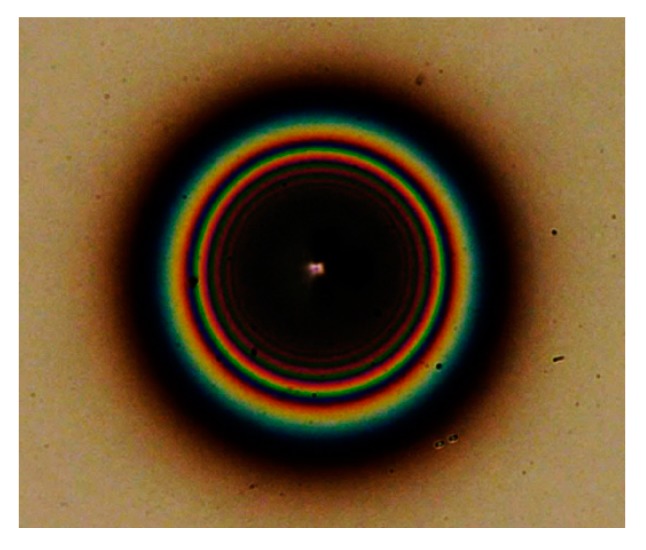
An optical microscope image for the film on Si wafer shown in Figure 8.

Figure 8a,b show SEM images of a deposited film on Si wafer from top view and side view, respectively, after 15 s operation at 200 μm gap length, 6.6 sccm C_2_H_2_ flow rate with 80 kPa inlet pressure, 1000 V source voltage and 19 μA initial discharge current. Figure 9 shows an optical microscope image for the deposited film. The deposited film was not uniform but circularly-shaped with a diameter of 220 μm. As shown in Figure 8b, the deposited film was fairly thick as directly recognized from the tilted angle observation. From the multiple concentric rings for optical interference in the film appeared in Figure 9, the profile was a symmetric shallow mountain shape. 

**Figure 10:**
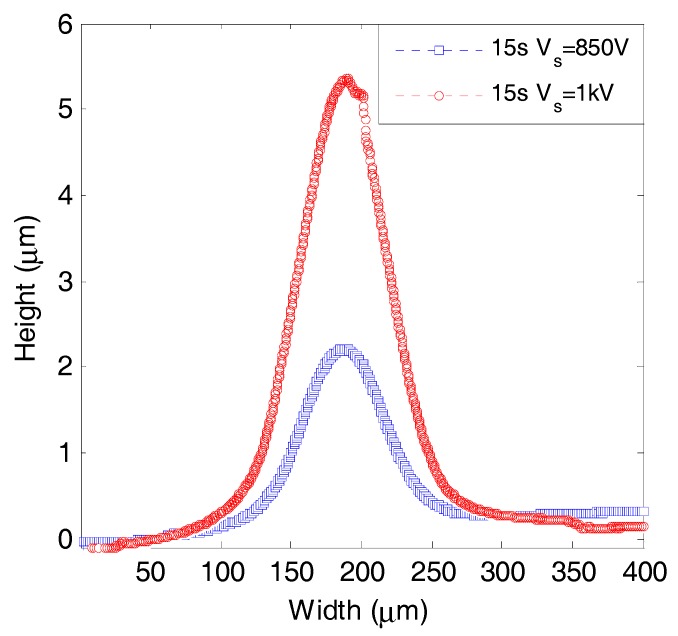
Thickness profiles of deposited films for 15 s at 850 and 1000 V source voltage.

Figure 10 shows thickness profile of films on Si wafer deposited for 15 s at 200 μm gap length, 6.6 sccm C_2_H_2_ flow rate with 80 kPa inlet pressure, 850 or 1000 V source voltage with 13 or 19 μA initial current, respectively. The sizes of the deposited areas were evaluated as 175 and 220 μm for 850 and 1000 V, respectively. With increasing the source voltage, the deposition area was enlarged. As mentioned in the previous section, when the central area was fully covered with insulating film, the surrounding area of the Si wafer worked as a cathode. In the surrounding area, however, it required a higher voltage for discharge due to the longer pass and the dramatically decreased pressure. By applying a higher source voltage, the gas discharge can be sustained at farther distance from the center, resulting in a larger deposition area. 

The deposition rates at the central position were evaluated as 0.15 and 0.33 μm/s for 850 and 1000 V source voltage, respectively. The deposition rates of this micro plasma jet were much higher than those by the conventional PECVD method by almost 10 times [21]. From the discussion in the previous section, the plasma would be focused in the central area at the beginning of discharge due to higher gas pressure than the surrounding area. Due to progress in deposition at the central area, the plasma profile would move to the surrounding area. It can be speculated that the deposition rate is not constant at each position, but gradually changes with the progress of deposition. 

It was confirmed that a symmetrical profile thick film was automatically achieved within a short duration by the presented micro gas jet configuration without any complex design [22]. The smooth, symmetric, and fast film deposition will be advantageous for application to electronic devices. 

**Figure 11:**
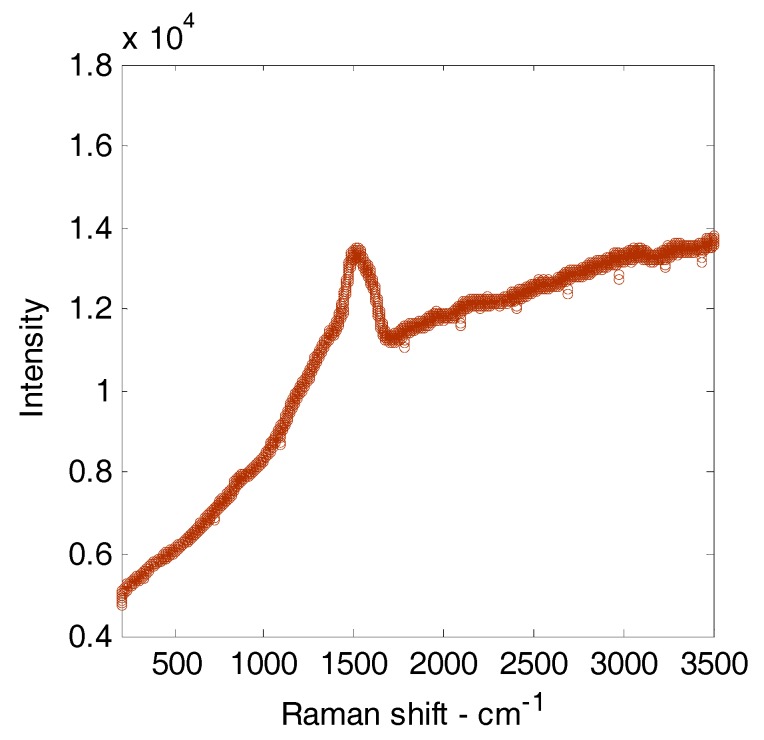
A typical Raman spectrum for a film deposited for 15 s at 200 μm gap length, 6.6 sccm C_2_H_2_ flow rate, 80 kPa inlet pressure, and 1000 V source voltage.

Figure 11 shows a typical Raman spectrum for a film deposited for 15 s at 200 μm gap, 6.6 sccm C_2_H_2_ flow rate, 80 kPa inlet pressure, and 1000 V source voltage. The Raman spectrum shows a peak of G band and D band, which originate from hydrogenated amorphous carbon film (a-C:H), at around 1521 cm^−1^ and 1367 cm^−1^, respectively. The ratio of the D and G band intensities (I_D_/I_G_ ratio) indicated that a-C:H film was structurally similar to DLC films [23]. At this experimental condition, the I_D_/I_G_ ratio was approximately 0.8517, which was smaller than that of some conditions of a-C:H films grown by dip-coating, pulsed laser deposition, and PECVD methods of [23,24]. 

**Figure 12:**
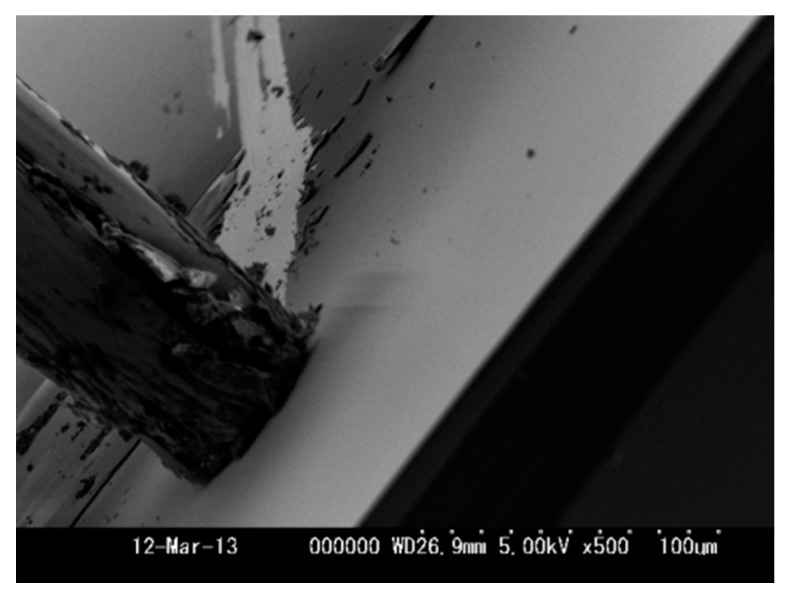
An SEM image for the hardness and adhesion test using a W needle.

After that, the film hardness and adhesion to the Si wafer were simply examined by scratching with a tungsten (W) needle in SEM as shown in Figure 12. The end of needle was a round column, 30 μm in diameter. With observation of the SEM image, the needle was pressed vertically on the film for indentation test and was scratched horizontally on the surface. By pressing on the film, the smooth surface was not damaged due to sufficient hardness for the compressive stress. By scratching on the surface, however, the film was peeled from the interface of the Si wafer. Since the deposition parameters—such as the operating pressure, C_2_H_2_ concentration, and depositing time—have strongly influenced on the quality of thin-film, how to improve the film quality by our proposed method will be studied in further work.

## 4. Conclusions

A DC micro plasma jet in a vacuum environment of an SEM chamber has been applied to local CVD process with in situ observation. By using an orifice-shaped gas nozzle as an anode, a high density gas jet with a micro diameter was successfully formed in vacuum with a gas flow rate enough to keep the high vacuum level. Hydrogenated amorphous carbon film (a:C-H) was locally deposited on the cathode of Si wafer from C_2_H_2_ source gas. The voltage and current of micro plasma discharge rapidly changed with operation time due to the local deposition of insulating film on the cathode. The profile of the film thickness was symmetrical and mountain shaped and the hardness was fairly high while the adhesion to the Si wafer was poor. The deposition rate at the center of the jet was quite large, for example 0.33 μm/s.
